# Evaluation of a diluted lipid emulsion solution as a lubricant for improved peripherally inserted central catheter guidewire removal in a neonatal population

**DOI:** 10.1186/s12887-022-03119-2

**Published:** 2022-01-31

**Authors:** Matheus F. P. T. van Rens, Ratheesh Paramban, Airene L. V. Francia, Prem Chandra, Mohamad Adnan Mahmah, Ulrich H. Thome, Mohammad A. A. Bayoumi, Timothy R. Spencer

**Affiliations:** 1 Neonatal Intensive Care Unit, Women’s Wellness and Research Center, Hamad Medical Corporation, Doha, Qatar; 2 Academic Health System, Hamad Medical Corporation, Doha, Qatar; 3 Division of Neonatology, University Hospital for Children and Adolescents, Women’s and Children’s Hospital, Leipzig, Germany; 4 Global Vascular Access LLC, Scottsdale, AZ USA

**Keywords:** Neonate, Peripherally inserted central catheter, Guidewire, Lipid emulsion, Complications, Malposition

## Abstract

**Background:**

Medical management of neonates is often established upon safe and reliable vascular access, frequently utilized to provide physiological monitoring, parenteral and supportive treatments, and diagnostic and/or procedural purposes. For this, peripherally inserted central catheters (PICCs) are often used to provide safe vascular access and infusion-related therapies in the neonatal intensive care (NICU) setting.

**Purpose:**

Difficult PICC guidewire removal is understood to cause catheter damage, causing luminal rupture or possible breakage of the catheter or guidewire itself. The aim of this study was to assess and compare the incidence of therapy failures with use of a preflush fluid using normal saline (NSS) versus a diluted lipid solution (DLS) prior to device insertion, to assist with guidewire removal and prevent unnecessary catheter damage.

**Method and setting:**

A retrospective, observational study was performed in the Neonatal Intensive Care Unit (NICU) of the Women’s Wellness and Research Centre, Hamad Medical Corporation, Qatar. This single site study included 507 neonates who required intravenous therapy administered via a PICC during the study period.

**Results:**

Results demonstrated the use of a diluted lipid solution preflush (DLS) resulted in significantly lesser failures, when compared with the control group (NSS). This highlights a clinical significance after adjusting for day of insertion, gestational age, birth weight and catheter type.

**Conclusion:**

DLS preflush demonstrated a benefit over the use of a NSS preflush to enhance PICC guidewire removal in neonatal patients in the NICU. The risk for development of maintenance-related complications leading to premature device removal decreased significantly if the DLS preflush was used. During the study period, no complications related to the use of a lipid preflush solution were identified.

**Implications for practice and research:**

This may be the first study published investigating and supporting guidewire removal enhancement by using a diluted lipid/saline preflush solution. When the requirement for vascular access is most pertinent in the neonate, using a diluted lipid preflush may provide an effective method to assist in guidewire removal to prevent malposition and vascular device complications in the neonatal population.

## Background

Medical management of neonates is often established upon safe and reliable vascular access needs. The need for safe and reliable vascular access may be related to physiological monitoring (arterial or central venous pressure), parenteral treatments (antibiotics, analgesia, inotropes), supportive therapy (intravenous nutrition, blood products administration, ECMO) and diagnostic or procedural purposes (power contrast injection for MRI/CT, cardiac catherization) [1, 2]. Vascular access devices (VADs) such as peripherally inserted central catheters (PICCs) play a vital role in the management with this patient population.

Due to unique developmental characteristics, preterm infants are more susceptible to iatrogenic harm arising from vascular cannulation and its related complications. It is widely accepted that healthcare staff should take quality measures to prevent, detect, promptly treat, and mitigate any of these risks. Internationally, most neonatal units have implemented bundles of measures to reduce and manage risks associated with vascular access. One key element of these ‘care bundles’ is directed towards prevention of device-related complications, e.g., large catheters placed in small veins, associated pain from frequent replacement of peripheral cannulas, chemical-related vessel trauma from parenteral nutrition solutions or drugs that have irritant or vesicant properties [3].

In the neonatal intensive care (NICU) setting, PICCs have had reported complication rates between 0.05 to 3% [4]. Generally, PICCs are commonly associated with a reduced incidence of complications compared to short peripheral catheters [5]. Despite these advantages, PICCs may still be associated with complications such as device occlusion, infection, thrombosis, tip malposition, and potential catheter damage [4, 5], resulting in interrupted treatments, failed venous access and greater resource consumption with catheter replacement [6, 7].

Catheter damage, that causes leaking and embolization of the catheter itself, may be related to difficult guidewire removal during the insertion procedure, considering the nature of such small devices [8]. The inability to remove the guidewire possibly requires removing the device and reinserting a new catheter. The etiology of this phenomena can be explained by a guidewire with approximately the same diameter as the actual inner diameter of the PICC, resulting in the wire sticking or getting caught up to the catheter’s internal wall. Despite generous pre-flushing of the catheter and guidewire using a normal saline solution (NSS) prior to insertion, removing the internal PICC guidewire once the device is insitu, can be challenging. Increased withdrawal resistance on removal of the guidewire, even after correct catheter tip position is confirmed by x-ray or ultrasound, may lead to tip malposition (tip will be pulled out of the superior vena cava/cavo-atrial junction), but also potentially cause catheter leakage or breakage (shear or tearing forces on the wall of the catheter) [9]. Several interventions are described to decrease guidewire resistance during removal [8]. Reduction of any tension before attempting to remove the guidewire can be achieved by straightening and untwisting the parts of the PICC still outside the body [8]. In this study setting, it was reported that PICC guidewire removal-related complications occurred in 11/450 (2.4%) of all PICC insertions. Despite the relatively low numbers, the clinical impact to the patient of device malposition or damage must be considered. Recent evidence from large scale studies in neonatal populations regarding guidewire removal is lacking and largely absent.

A novel approach to overcome difficult guidewire removal is to use a diluted lipid solution (DLS) as a lubricant (Thome, personal correspondence) [10]. Although this has been used successfully in practice, particularly with intractable removals, there are potential safety concerns such as lipid emboli and risk of infection [11, 12]. Further empirical study is required to quantify these risks and until this evidence is available, this approach should be used with careful reflection and an established clinical benefit [8].

This study aims to highlight patient- (gestation, weight) and VAD characteristics (type, diameter and length) and assess the outcomes (guidewire removal, therapy failure) between conventional normal saline solution (NSS) and diluted lipid solution (DLS) as a lubricant to enhance difficult guidewire removal amongst a neonatal population.

## Methods

### Ethical approval

The study design and procedures (MRC-01-20-890) were approved by the local Institution Review Board (IRB). As the data source was anonymized, the local IRB committee determined the study an ‘observational chart review’ and that participant consent was not required.

### Design and setting

A retrospective, cross-sectional study, using routinely collected anonymized intravenous device data was performed from January 2019 through July 2020. The primary outcome of interest was the occurrence of therapy failure (e.g., leaking, breakage) in relation to PICC guidewire removal leading to any unplanned device removal prior to completion of therapy. The study was performed in the 112-bed NICU of the Women’s Wellness and Research Centre (WWRC) of Hamad Medical Corporation (HMC), Doha, Qatar.

### Patient and public involvement statement

Study participants, nor parents were not involved in the design, conduct or reporting of this study. Retrospective data were retrieved from the electronic data management system of the facility.

### Participants and sample size

Infants who were admitted to the NICU and who needed intravenous therapy through a 1Fr./28G or 2Fr./24G PICC were included in this study. Participants were excluded from the sample if the data collection was incomplete or whenever the data was related to the use of other devices e.g., peripheral IV catheters, umbilical venous catheters (UVC) or PICCs exceeding 2Fr./24G.

### Procedure

**Fig. 1:**
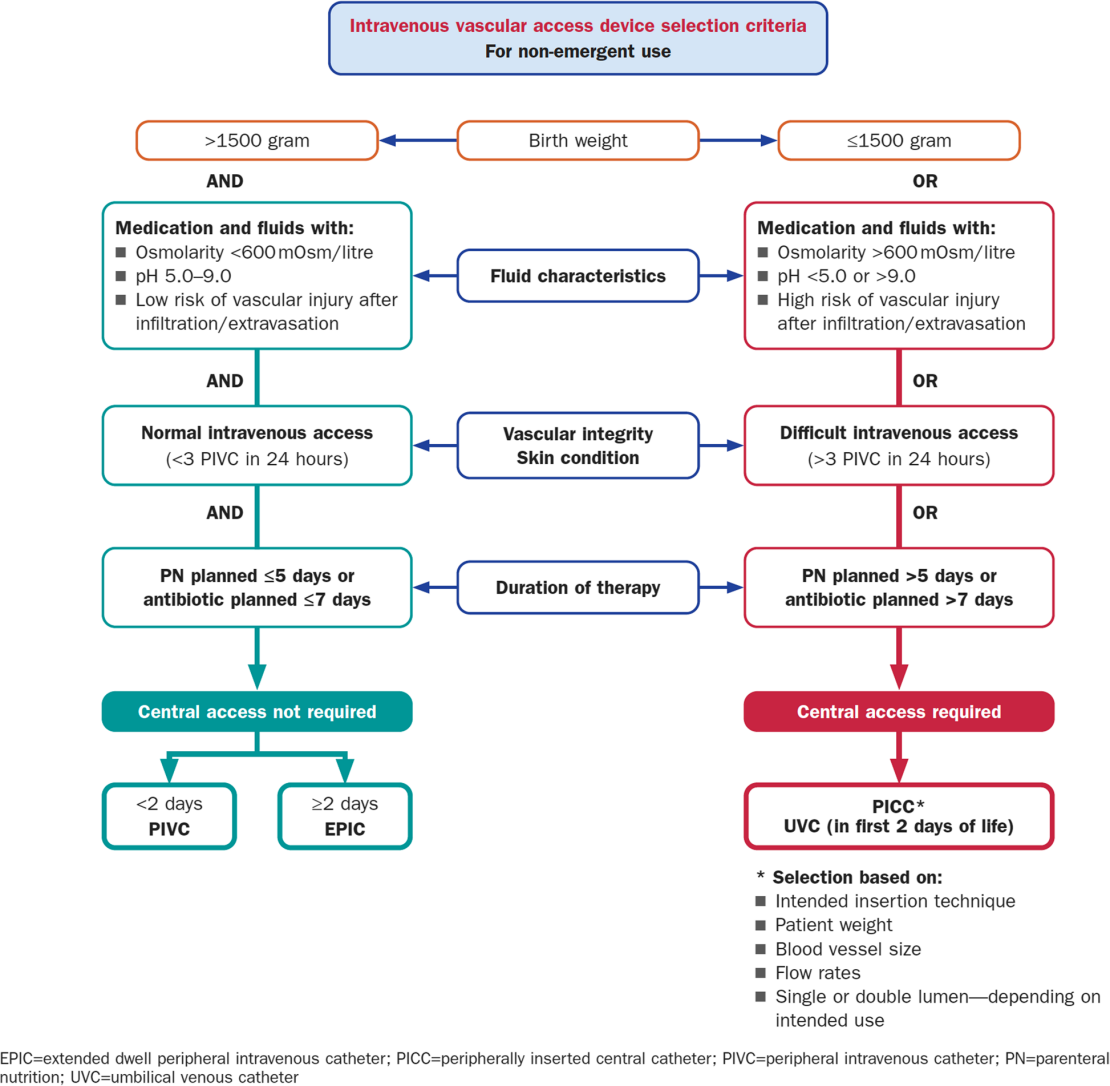
Vascular Access Device Algorithm

During the patient assessment stage, the vascular access team follows a locally developed mnemonic, the “5Rs for Vascular Access” i.e., the Right device, for the Right vein, with the Right therapy, for the Right duration, for the Right patient, as described in a similar concept by Steere et al. [13] Venous cannulation is strictly performed under guidance of the local hospital policy, based on current evidence-based recommendations and guidelines [14, 15]. In this study setting, a PICC insertion is routinely performed by doctors and nurses from the neonatal vascular access team (neoVAT). The selection of appropriate veins are performed using near-Infra-Red (n-IR) technology for vein visualization (Christie Medical Holdings, Lake Mary, Florida, USA). In this facility’s practice, the choice for a central vascular access device is based upon the abovementioned 5Rs, with the required duration, fluid characteristics (pH and osmolarity) and patient characteristics (body weight and/or a known history of difficult vascular access). The 5Rs for Vascular Access-concept is represented in Fig. 1 and are based upon current standards of care [14, 15] and also considers product compatibility, hospital purchasing decisions and practitioner consensus.

All preflush solutions were prepared at the bedside, under sterile conditions. For the DLS, a 1 ml lipid 20% was diluted with 9 ml normal saline, the NSS was provided as prefilled 10 ml syringe flushes. Preflushes of NSS and DLS were provided alternated for the daily PICC insertions. Note: all PICCs were checked and flushed outside the patient and before the actual insertion procedure, to avoid excessive infusion of the preflush solution. Once the PICC was successfully inserted, the guidewire was then removed without any further flushing with either the NSS or DLS solutions. Total filling volumes of these PICC’s are extremely small and would vary between a maximum of 0.09-0.12 ml, depending on type and length of the device. During guidewire removal, a small amount of the DLS volume is removed due to adhesion of the fluid to the guidewire.

### Measurements and data collection

Patients demographics and baseline data included sex, gestational age at birth in weeks and days, birth weight, and current body weight in grams. Data regarding the procedure of intravenous cannulation were the date and time of cannulation, including the number of attempts to successful cannulation, cannulated extremity, used type and size of vascular access device, indication for intravenous treatment, type of preflush used, any noted resistance during guidewire removal, date and time of PICC removal, total PICC dwell time in days, and the reason for PICC removal.

### Statistical analyses

A total of 507 cases were retrospectively collected between January 2019 through to July 2020. Descriptive statistics were used to summarize and determine the sample characteristics and distribution of study participants data. The normally distributed data and results were reported with mean and standard deviation (SD); the remaining results reported with median and inter-quartile range (IQR). Categorical data were summarized using frequencies and proportions. Associations between two or more qualitative data variables were assessed using Chi-square (χ2) test or Fisher Exact test as appropriate. Quantitative data between the two independent groups were analyzed using unpaired t or Mann Whitney U test as appropriate. Univariate and multivariate logistic regression analysis (controlling and adjusted for potential predictors and confounders such as type of preflush, gender, resistance of the guidewire, days at insertion, birthweight, gestation at birth, number of attempts, extremity of cannulation, catheter characteristics, indication for intravenous therapy, indwell time) were applied to determine and assess the associations and predictive values of predictors and confounders stated above with binary outcome variable risk for failure of intravenous access devices. For multivariate logistic regression models, predictor variables were considered if statistical *P* < 0.10 level in univariate analysis or if determined a priori to be clinically important. The results of logistic regression analyses were presented as odds ratio (OR) with corresponding 95% CI. Receiver operating characteristic curve (ROC) was computed and constructed to evaluate and assess predictive accuracy and discriminative ability of the developed logistic regression model (based on the predicted probabilities) using potential significant variables found in the multivariate logistic regression model. All *P* values presented were two-tailed, and P values < 0.05 was considered as statistically significant. All Statistical analyses were done using statistical packages SPSS version 27.0 (Armonk, NY: IBM Corp) and Epi-info (Centers for Disease Control and Prevention, Atlanta, GA) software.

## Results

Study demographics as gender, age at PICC insertion, gestational age and birthweight, as well as chosen extremity for device insertion are presented in Table 1. The data demonstrated that 60.2% of patients received the DLS, and 39.8% received the NSS preflush. Most catheter insertions were in the lower extremities (420/507; 82.8%) in both type of preflush solutions. The day of life on which the PICC insertion took place was an average of 7.61 days (+ 17.0) with median 3 days (inter-quartile range 2 to 5) and there was no significant difference observed between both preflush groups. More detailed data regarding the type of flush showed a lower percentage in the NSS group for neonates 32-36wks gestation (NSS 16.3% vs DLS 19.3%) and in the birthweight category of 1500-2499 g (NSS 19.8% vs DLS 24.6%). However, this showed a reversed percentage for neonates with a birthweight > 2500 g (NSS 14.9% vs DLS 6.2%) and a gestational age of >37wks (NSS 12.4% vs DLS 6.6%).

**Table 1:** Demographic Patient Factors

Type of Flush							
	Total (***n*** = 507)		Normal Saline (NSS) (***n*** = 202)		Diluted Lipid (DLS) (***n*** = 305)		***P***-value
	***n***	%	***n***	%	***n***	%	
Gender							
Male	277	54.6%	125	61.9%	152	49.8%	0.008*
Female	230	45.4%	77	38.1%	153	50.2%	
Days of life at insertion	7.61 ± 17.0		7.34 ± 19.0		7.78 ± 15.6		0.770
Mean ± SD (median, IQR)	(3, IQR 2, 5)		(3, IQR 2, 5)		(2, IQR 2, 5)		
1-50 days	490	96.9%	197	97.5%	293	96.1%	
51-100 days	13	2.6%	3	1.5%	10	3.3%	
101-150 days	3	0.6%	1	0.5%	2	0.7%	
≥ 151 days	1	0.2%	1	0.5%	0	0.0%	
GA at birth (days), Mean, SD	212.0 ± 28.2		213.64 ± 30.0		210.97 ± 26.8		0.026*
23-27wks	148	29.2%	59	29.2%	89	29.2%	
28-31wks	222	43.8%	85	42.1%	137	44.9%	
32-36wks	92	18.1%	33	16.3%	59	19.3%	
≥ 37wks	45	8.9%	25	12.4%	20	6.6%	
Birth Weight (gm) Mean, (SD)	1421.7 ± 688.1		1494.5 ± 762.1		1373.4 ± 631.1		0.052
≤ 999 g	133	26.2%	49	24.3%	84	27.5%	
1000-1499 g	210	41.4%	83	41.1%	127	41.6%	
1500-2499 g	115	22.7%	40	19.8%	75	24.6%	
≥ 2500 g	49	9.7%	30	14.9%	19	6.2%	
Limb Extremity							
Upper	87	17.2%	41	20.3%	46	15.1%	0.130
Lower	420	82.8%	161	79.7%	259	84.9%	

*IQR* Inter-quartile range

A majority of all PICC (422/507; 83.2%,) were inserted based on the required duration for extended total parenteral nutrition (TPN). The results of these insertion and catheter details are reflected in Table 2. The 1Fr./24G PICC were the most used type of catheter in the study population, accounted for (456/507; 91.7%) of all insertions. A first-time success (FTS) rate was observed in (361/507; 71.2%) and FTS was reported more frequently in the lipid solution group (224/361; 62.0%) vs the saline group (137/361; 38%).

**Table 2:** Insertion and Catheter Details

Type of Flush							
	Total (***n*** = 507)		Normal Saline (NSS) (***n*** = 202)		Diluted Lipid (DLS) (***n*** = 305)		***P***-value
	***n***	%	***n***	%	***n***	%	
Reason for Insertion							0.16
Antimicrobial Therapy	17	3.4%	5	2.5%	12	3.9%	
PN Therapy	422	83.2%	165	81.7%	257	84.3%	
Fluid characteristics^a^	23	4.5%	14	6.9%	9	3.0%	
Difficult Vascular Access	45	8.9%	18	8.9%	27	8.9%	
Catheter Type							
2 Fr. PICC 15 cm	13	2.6%	6	3.0%	7	2.3%	0.139
2 Fr. PICC 30 cm	29	5.7%	17	8.4%	12	3.9%	
1 Fr. PICC 15 cm	12	2.4%	7	3.5%	5	1.6%	
1 Fr. PICC 20 cm	296	58.4%	111	55.0%	185	60.7%	
1 Fr. PICC 30 cm	157	31.0%	61	30.2%	96	31.5%	
Number of attempts							0.374
1	361	71.2%	137	67.8%	224	73.4%	
2	94	18.5%	39	19.4%	55	18.0%	
3	47	9.3%	23	11.4%	24	7.9%	
4	5	1.0%	3	1.5%	2	0.7%	
Guidewire resistance during removal							< 0.001*
Neutral	59	11.6%	26	12.8%	33	10.85	
Negative	358	70.5%	87	43.1%	271	88.9%	
Positive	90	17.7%	89	44.1%	1	0.3%	

*PN* Parenteral nutrition; ^a^ based upon pH and osmolality; *-

For this study therapy failure related to the chosen type of preflush was observed. Table 3 shows a majority (401/507; 88.5%) of PICC were electively removed after the therapy was completed. When neonates were transferred to another hospital or expired (54/507; 10.7%), the team considered such cases as lost to follow up (local administrative censoring), these patients were excluded for therapy failure statistics. More successful or elective removals were within the DLS group (247/272; 90.8%) vs (154/181; 85.1%). All non-elective removals of PICCs occurred because of complications*.* Moreover, failure of therapy was more common in NSS group (27/181; 14.9%) than when using the DLS flush (25/272; 9.2%). The reasons of therapy failure like breaking or leaking of the PICC, catheter-related complications, extravasation or infiltration, and suspected sepsis were commonly reported in NSS group (22/181; 12.2%) vs (14/272; 5.2%), whereas complications due to maintenance and phlebitis were more frequently observed in the DLS group (11/305; 4.0%) vs (5/202; 2.8%).

**Table 3:** Data Representing the Different Factors of VAD Removal

Type of Flush							
	Total (***n*** = 507)		Normal Saline (NSS) (***n*** = 202)		Diluted Lipid (DLS) (***n*** = 305)		***P***-value
	***n***	%	***n***	%	***n***	%	
Reason for Removal							
Administrative censoring^a^	54	10.7%	21	10.4%	33	10.8%	0.260
Therapy success (elective removal)	401	88.5%	154	85.0%	247	90.8%	
Therapy failure^b^	52	11.5%	27	15.0%	25	9.2%	
Breakage/leakage of material	*5*	*1.1%*	*4*	*2.2%*	*1*	*0.4%*	
Catheter-related complications^c^	*14*	*3.1%*	*9*	*5.0%*	*5*	*1.8%*	
Extravasation/Infiltration	*2*	*0.4%*	*1*	*0.6%*	*1*	*0.4%*	
Maintenance-related complications^d^	*13*	*2.9%*	*4*	*2.2%*	*9*	*3.3%*	
Phlebitis	*3*	*0.7%*	*1*	*0.6%*	*2*	*0.7%*	
Suspected sepsis	*15*	*3.3%*	*8*	*4.4%*	*7*	*2.6%*	
Dwell Time (days) Mean, (SD)	12.97 ± 8.1		12.53 ± 8.2		13.26 ± 8.0		0.320

^a^Administrative censoring = death (other causes than CLABSI) and neonates transferred out. ^b^For therapy failure administrative censoring is excluded

^c^Catheter related complications are defined as tip malposition, leaking, breakage of the catheter. ^d^Maintenance related complications are defined as accidental removal and occlusion

Based on the results presented in Table 4, birthweight in grams, gestation in weeks, number of attempts and catheter dwell time have significant effect on the likelihood of failure of therapy. Results indicate that increasing birthweight ≥2500 g and gestation at birth ≥37 weeks were associated with an increased likelihood of failure of therapy. However, birthweight 1500-2499 g (unadjusted OR 0.33; 95 CI 0.12, 0.93, *P* = 0.036) and 32-36 weeks of gestation (unadjusted OR 0.27, 95 CI 0.09-0.76), *P* = 0.014) were found to be significantly associated with reduced risk of failure of therapy. Increasing time of the PICC in situ were observed to be significantly associated with a reduction in the likelihood of failure of therapy. Results show that a fourth attempt to successful cannulation compared to the first attempt to successful cannulation has a significant effect on the increased risk of likelihood of failure of therapy (unadjusted OR 9.48; 95% CI 1.29-69.7, *P* = 0.027). Furthermore, fourth attempts of successful cannulation were around nine times more likely to have failure of therapy than first attempts of successful cannulation. The dwell time showed an inverse significant effect whereby the likelihood to failure was reduced when compared between 7 to 27 days. Specifically, > 7 to 14 days (unadjusted OR 0.18; 95% CI 0.09-0.35, *P* = < 0.001), > 14 to 21 days (unadjusted OR 0.18; 95% CI 0.07- 0.45, *P* = < 0.001), and > 21 to 27 days (unadjusted OR 0.11; 95% CI 0.01- 0.88, *P* = 0.037) were with significant effect to risk of failure of therapy.

**Table 4:** Binary Logistic Regression Analyses with Factors Affecting the Risk for Failure of Intravenous Access Devices

Variable	Therapy failure, n (%)	Unadjusted Odds ratio (OR)	95% CI (for OR)	***P***-value
**Gender**				
Female	21 (10.2%)	1.0 (reference)		
Male	31 (12.5%)	1.25	0.70, 2.25	0.454
**Type pre-flush**				
Lipid (DLS)	25 (9.2%)	1.0 (reference)		
Normal saline (NSS)	27 (14.9%)	1.73	0.97, 3.09	0.063
**Guidewire resistance during removal**				
Neutral	8 (16.7%)	1.0 (reference)		
No resistance	33 (10.1%)	0.56	0.24, 1.30	0.178
Resistance	11 (14.1%)	0.82	0.31, 2.12	0.696
**Birthweight (grams)**				
≤ 999 g	16 (13.6%)	1.0 (reference)		
1000-1499 g	20 (10.4%)	0.74	0.37, 1.50	0.403
1500-2499 g ≥2500 g	5 (4.9%)	0.33	0.12, 0.93	0.036*
≥ 2500 g	11 (26.8%)	2.34	0.98, 5.57	0.055
**Gestation at birth (weeks)**				
23-27 weeks	15 (14.4%)	1.0 (reference)		
28-31 weeks	21 (11.1%)	0.74	0.36, 1.51	0.410
32-36 weeks	5 (4.3%)	0.27	0.09, 0.76	0.014*
≥ 37 weeks	11 (25%)	1.98	0.83, 4.74	0.126
**Number of attempts**				
1	31 (9.5%)	1.0 (reference)		
2	14 (16.9%)	1.92	0.97, 3.81	0.060
3	5 (12.2%)	1.32	0.48, 3.60	0.591
4	2 (50.0%)	9.48	1.29, 69.7	0.027*
**Side of cannulation**				
Left	12 (11.1%)	1.0 (reference)		
Right	40 (11.6%)	1.05	0.53, 2.08	0.891
**Extremity**				
Lower extremity	39 (10.3%)	1.0 (reference)		
Upper extremity	13 (17.3%)	1.82	0.92, 3.61	0.085
**Catheter characteristics**				
2 Fr. PICC 15 cm	3 (27.3%)	1.0 (reference)		
2 Fr. PICC 30 cm	6 (21.4%)	0.73	0.15, 3.62	0.697
1 Fr. PICC 15 cm	1 (9.1%)	0.27	0.02, 3.08	0.290
1 Fr. PICC 20 cm	29 (10.9%)	0.33	0.08, 1.31	0.114
1 Fr. PICC 30 cm	13 (9.4%)	0.28	0.07, 1.18	0.082
**Indication for intravenous therapy**				
Antimicrobial Therapy	2 (15.4%)	1.0 (reference)		
TPN Therapy	38 (10%)	0.61	0.13, 2.87	0.534
Fluid characteristics	5 (25%)	1.83	0.30, 11.26	0.513
Difficult Vascular Access	7 (17.1%)	1.13	0.20, 6.28	0.887
**Indwell time catheter**				
< =7 days	23 (31.1%)	1.0 (reference)		
> 7 to 14 days	18 (7.4%)	0.18	0.09, 0.35	< 0.001**
> 14 to 21 days	7 (7.5%)	0.18	0.07, 0.45	< 0.001**
> 21 to 27 days	1 (4.8%)	0.11	0.01, 0.88	0.037*
> 27 days	3 (15.0%)	0.39	0.10, 1.47	0.164

*CI* Confidence interval; * Significant at 0.05 level of significance; **significant at 0.01 level of significance

The multivariate logistic regression analysis showed that both duration of gestation (weeks) indwell time catheter (days) were significantly associated with the risk of failure to therapy after controlling and adjusting potential confounders and predictors as shown in Tables 5 and 6 The association between increasing duration of gestation in 32-36 weeks (adjusted OR 0.12; 95% CI 0.04- 0.38, *P* = < 0.001) and the dwell time was associated with a reduction in the likelihood of failure of therapy when compared between < 7 days to other categories. Specifically, > 7 to 14 days (adjusted OR 0.14; 95% CI 0.06-0.30, *P* = < 0.001), > 14 to 21 days (adjusted OR 0.10; 95% CI 0.04-0.29, *P* = < 0.001), and > 21 to 27 days (adjusted OR 0.06; 95% CI 0.01-0.49, *P* = 0.009) and > 27 days (adjusted OR 0.21; 95% CI 0.05-0.89, *P* = 0.034) demonstrated significant effect at risk of failure. The differences between the two types of preflush to the likelihood of failure of therapy was considered statistically significant at the 0.10 level of significance.

**Table 5:** Multiple Logistic Regression Analyses with Factors Affecting the Risk for Failure of Intravenous Access Devices

Factors	Therapy failure n (%)	Adjusted Odds ratio (OR)	95% CI for OR	***P***-value
**Gestation at birth (weeks)**				
23-27 weeks	15 (14.4%)	1.0 (reference)		
28-31 weeks	21 (11.1%)	0.53	0.24, 1.16	0.114
32-36 weeks	5 (4.3%)	0.12	0.04, 0.38	< 0.001**
≥ 37 weeks	11 (25%)	0.61	0.21, 1.78	0.363
**Indwell time catheter**				
< =7 days	23 (31.1%)	1.0 (reference)		
> 7 to 14 days	18 (7.4%)	0.14	0.06, 0.30	< 0.001**
> 14 to 21 days	7 (7.5%)	0.10	0.04, 0.29	< 0.001**
> 21 to 27 days	1 (4.8%)	0.06	0.01, 0.49	0.009*
> 27 days	3 (15.0%)	0.21	0.05, 0.89	0.034*

*CI* Confidence interval; * Significant at 0.05 level of significance; **significant at 0.01 level of significance

**Table 6:** Association between CLABSI and Type of Pre-flush

Preflush Type				***P***-value
		Lipid (DLS)	Saline (NSS)	
**CLABSI**	Positive	3 (1%)	0 (0%)	0.434*
	Negative	302 (99%)	202 (100%)	

* Chi-Square-Fisher Exact test

**Fig. 2:**
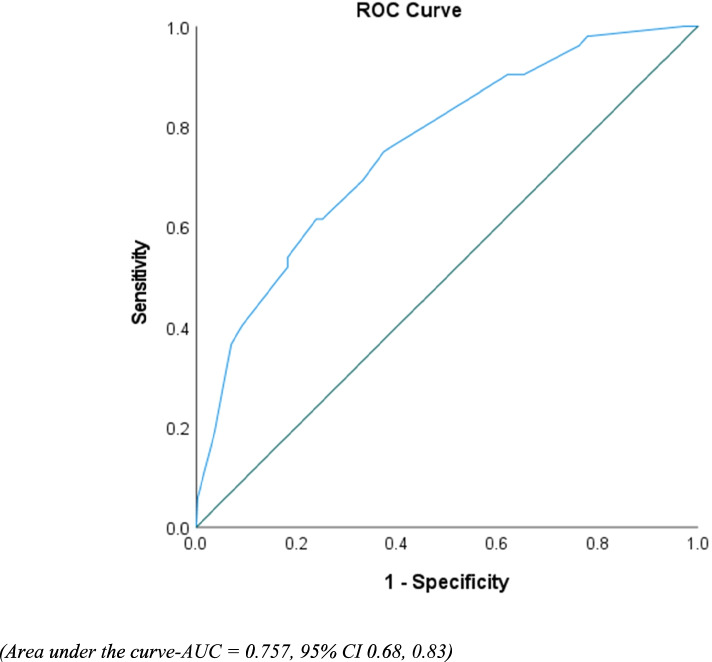
Receiver operating characteristic curve (ROC) to evaluate and assess predictive accuracy of the developed logistic regression model (using the predicted probabilities)

Therefore, a computed prediction model was used to evaluate the discriminative ability of potentially significant predictors (observed in the developed multivariate logistic regression model) associated with risk of failure to therapy using ROC curve analysis. The value of area under the curve (AUC) observed was 0.757 (95% CI 0.68, 0.83), indicated that this developed regression model demonstrated an excellent fit (see Fig. 2).

Central line-associated bloodstream infections (CLABSI) and type of preflush was tested for association using a Chi square test, however this demonstrated no statistical significance between either group (*P* = 0.434).

## Discussion

The external catheter diameters used in this study were 0.7 mm (1Fr.) and 1.1 mm (2Fr.) for devices used in this study, as documented on the product lid stock. The total priming volume varied from 0.09 ml to 0.12 ml, depending on the chosen catheter type and size (Vygon catheter product insert). The actual priming volume would be significantly less due to the guidewire size displacing an unidentifiable volume inside the PICC lumen (this was not measured during this study).

There may be elevated risk of PICC and infusion therapy failures within the clinical setting of the NICU, which may negatively affect a neonate’s treatment and outcomes [7]. Failure of therapy, resulting in premature removal, occurred in 54/453 (11.47%) of participants (transferred and death excluded), and a reported complication incidence rate of 7.91/1000 device days. The most frequently reported therapy failure prompting device removal was a catheter-specific complication (tip malposition, leaking, breakage of the catheter). The risk of catheter-related complications was reduced in patients with use of the DLS preflush for PICC guidewire removal. The normal and shear-and-tear forces between two opposing surfaces have been investigated and studies show that lipids may play a significant role [16]. Lower forces of friction may result in the reduction of shear-and-tear forces and its impact on the material of the catheter lumen itself. This study suggests that use of a DLS may reduce the coefficient of friction more effectively than NSS, resulting in reduced shear-and-tear related complications. Understanding the impact of frictional forces during guidewire removal is important with fragile, small bore vascular access devices, and further investigation is still needed to acknowledge the impact this may have on these devices.

Neonatal patients are an extremely vulnerable population. Vascular access devices provide the mainstay of all parenterally administered therapies for these, often-high-risk, patients. Reliability of PICC insertion and maintaining safe infusion therapy practices are paramount. While there are currently only NSS flushes available to clinicians, there remains a responsibility to explore other safe and effective alternatives for assisting guidewire removal in order to minimize complications.

Intravenous lipids may often provide a more ideal medium for microbial growth based upon their relatively neutral pH (pH = 8) [17]. The infection prevention department at the study facility found no published evidence for not safely introducing use of a DLS during PICC guidewire removal. This study found that the reported CLABSI rate during the study period was not statistically significant between either groups and the microbiological safety with use of a DLS preflush to assist guidewire removal during PICC insertion in the NICU did not demonstrate or contribute to any negative infectious outcomes.

### Strength and limitations

To the authors knowledge, this is the first study of this kind to evaluate the effect of a lipid-enhanced catheter preflush solution to assist PICC guidewire removal in the neonatal population worldwide. All eligible neonates were included during the study period, with the larger sample size representative of the facility’s neonatal PICC use. This helped increase the power of the study’s findings, helping to minimize any selection bias and increase the generalizability of the findings to similar settings.

Despite its strengths, this research also has some limitations. This study was a single center, retrospectively collected dataset, and in contrast to controlled randomized studies, this method creates risk of selection bias. During this study, every infant with a successfully inserted PICC was included to minimize the risk of selection bias. Data outcomes were not available for neonates transferred out of the facility. Although this number was relatively small, patients lost to follow-up may have differing outcomes than those who were included in this analyzed population. Nonetheless, future randomized control trails should focus on the continuous introduction of novel and clinically beneficial strategies to improve infusion therapy outcomes.

## Conclusion

This study demonstrated a clinical benefit from the use of DLS preflush to enhance ease of PICC guidewire removal, reducing device-related complications and impacting the successful completion of infusion therapies. These included reduced risk of damage or rupture/breakage to the lumen, catheter tip malpositions, and suspected sepsis. The risk for the development of maintenance-related complications leading to premature device removal was decreased significantly if the preflush DLS was used. The number of events for CLABSI rate were homogenous between the DLS and NNS groups, show no statistical significance. When the use of PICC is considered important for safe and reliable vascular access, DLS as a preflush for assisted guidewire removal offers a relatively safe and effective process in the neonatal population.

As only small amounts of lipid emulsion were used during this study, no other complications related to the use of a lipid emulsion were identified. Further research is still required. Secondary outcomes evaluating the cost effectiveness regarding the role and involvement of the pharmacy department and the nursing process of distributing and administering the diluted lipid solution may provide additional supportive evidence.

Identifying vascular access-related challenges for neonatal populations consistently requires innovation and clinical developments when trying to impact patient and vascular access device-related outcomes. The use of the DLS for PICC guidewire removal in these extremely small catheters is an important step for clinicians who place and care for vascular access devices in neonates.

## Data Availability

The data that support the findings of this study are available from Matheus van Rens but restrictions apply to the availability of these data, which were used under license for the current study, and so are not publicly available. Data are however available from the authors upon reasonable request and with permission of Matheus van Rens (rolandvanrens@icloud.com).
